# Editorial: In the March 2017 issue

**DOI:** 10.1590/1980-57642016dn11-010001

**Published:** 2017

**Authors:** Ricardo Nitrini

**Affiliations:** 1 Editor-in-Chief

In the March 2017 issue we are publishing three reviews, nine original papers and one (or two) case report(s).

**Ianof and Anghinah** presented a short review on the value of the EEG or qEEG for the diagnosis and prognosis of mild traumatic brain injury. They found that although promising tools, further studies are still needed.

**Spenciere et al.** reviewed historical aspects of the Clock Drawing Test (CDT) focusing on the scoring systems. They reported that initially quantitative and semi-quantitative scoring methods were preferred but more recently, qualitative analyses have begun to be used for early diagnosis of dementia and for differential diagnosis of the dementia subtype.

**Haase and Alves** reviewed the moral-psychological and neuroevolutionary basis of political partisanship. Three main points deeply related to political partisanship were identified and discussed: moral emotions, personality traits and self-deception. Cognitive and neuroscientific research is increasingly contributing to elucidate the psychological and neuroevolutionary mechanisms underlying political partisanship.

**Rodrigues-Neto et al.** investigated the neuropathological findings in the entorhinal cortex of subjects aged 50 and over using immunohistochemistry. In a small sample, they detected that individuals with dementia have more than one kind of protein deposit.

**Gondim et al.** performed an epidemiological study in Northeastern Brazil to investigate the prevalence of functional cognitive impairment (FCI) in a community- dwelling elderly population. The prevalence of FCI was high and found to be associated with age and vascular risk factors.

**Paiva et al.** investigated the influence of age on focused visual attention using the Bells Test. When comparing individuals aged 40-59 with individuals aged 60-75 there were no significant differences between the age groups. Further studies should investigate healthy individuals older than 75.

**Yassuda et al.** investigated the performance of 240 healthy community-dwelling elderly with low education background on the Brief Cognitive Screening Battery (BCSB). This battery was designed to be less influenced by education. The delayed recall test of the BCSB was more influenced by age than education. The norms reported by this study are important for the diagnosis of cognitive impairment in low educated elderly.

**Borges-Lima et al.** described the performance of a community-based sample of elderly adults on basic cognitive tasks and instrumental activities of daily living, including information on depressive symptoms. As subjects were independent elderly individuals who participated in multiple physical, social, and cognitive activities, the results contribute to a better understanding of the clinical concept of healthy aging.

**Campanholo et al.** evaluated the influence of age, education, income and occupational activity on executive functions in a large sample of healthy individuals whose age ranged from 18 to 89 years. They found that age had a negative influence, whereas education and income had a positive influence, on executive function. No influence of occupational activity was detected.

**Almondes et al.** investigated the effects of a cognitive training program and sleep hygiene orientation program on executive function performance of healthy elderly. They found that both programs were effective for improving executive functions and sleep quality, but no further gains were observed when both programs were combined.

**Pureza and Fonseca** described the development program and evaluation of content validity of the CENA (Program for Educational Training on the Neuropsychology of Learning), with an emphasis on executive functions (EF) and attention. This program was designed to promote additional knowledge on childhood neurocognitive development to allow teachers to integrate EF stimulation into their professional practice.

**Cardoso et al.** described the construction processes and content validity of a preventive intervention program to stimulate the executive functions in Elementary School children within the school environment. The thorough description of the procedures to develop the program had the objective of allowing other researchers to recreate similar programs or to make changes to better adapt to their environment.

**Shigaeff et al.** reported a case of primary progressive aphasia (PPA) and discussed the differential diagnosis among other degenerative dementias. The final diagnosis was the logopenic variant of PPA where the differential diagnosis with other variants of PPA was also discussed.

**Ricardo Nitrini** Editor-in-Chief

